# Improved Stage Categorization of PTZ-Induced Kindling and Late Enhanced Neurogenesis in PTZ Kindled Mice

**DOI:** 10.22086/gmj.v8i0.1511

**Published:** 2019-06-02

**Authors:** Marzieh Shahpari, Hadi Aligholi, Mohammad Reza Namavar, Farzaneh Vafaee, Masoumeh Emamghoreishi

**Affiliations:** ^1^Department of Neuroscience, School of Advanced Medical Sciences and Technologies, Shiraz University of Medical Sciences, Shiraz, Iran; ^2^Clinical Neurology Research Center, Shiraz University of Medical Sciences, Shiraz, Iran; ^3^Histomorphometry and Stereology Research Center, Shiraz University of Medical Sciences, Shiraz, Iran; ^4^Department of Anatomical Sciences, School of Medicine, Shiraz University of Medical Sciences, Shiraz, Iran; ^5^Department of Pharmacology, School of Medicine, Shiraz University of Medical Sciences, Shiraz, Iran; ^6^Research Center for Psychiatry and Behavior Science, Shiraz University of Medical Science, Shiraz, Iran

**Keywords:** Neurogenesis, Kindling, Subventricular Zone, Pentylenetetrazole, Seizure Behavior

## Abstract

**Background:**

There is no universally accepted behavioral scoring to define the early development of pentylenetetrazole (PTZ) kindling. Therefore, studies investigating alterations of neurogenesis in the PTZ model were mainly focused on full kindled animals rather than early stages of kindling. This study aimed to determine an appropriate behavioral index for categorizing stages of PTZ kindling progress and to evaluate neurogenesis during PTZ kindling.

**Materials and Methods:**

Twenty-four mice were intraperitoneally injected with a sub-convulsive dose of PTZ (40mg/kg) every other day until they became full kindled. The first occurrence of different seizure behaviors and their durations were recorded during kindling development, and the different stages of kindling were categorized. Neurogenesis was evaluated in the lateral subventricular zone (SVZ) at each stage of kindling by immunofluorescence staining.

**Results:**

First occurrence of restlessness, motionless staring, hind limb tonic extension, Straub’s tail, myoclonic jerk, and tonic-clonic were sequentially observed in more than 80% of animals with increasing PTZ injections. The duration of the myoclonic jerk was significantly longer than the other seizure behaviors. The significantly higher percentage of BrdU-positive cells was found in SVZ of mice showing tonic-clonic in comparison to other seizure behaviors.

**Conclusion:**

A hierarchy behavior was observed during the kindling process when considering the first occurrence of seizure behaviors. We defined the first occurrence of restlessness, motionless, hind limb tonic extension and Straub’s tail behaviors as an early phase, myoclonic jerk as a borderline phase and tonic-clonic as a late phase of PTZ-induced kindling. Our results indicated an enhanced SVZ neurogenesis at the late phase of kindling.

## Introduction

Epilepsy, the fourth most common neurological disorder worldwide, is characterized by recurrent electrical discharges spreading to cortices, which are accompanied by neuropathological changes [1]. Temporal lobe epilepsy (TLE) is the most common type of epilepsy [2]. Alterations in neurogenesis in the subgranular zone of the hippocampal dentate gyrus and the subventricular zone (SVZ) of the forebrain lateral ventricles have been reported in TLE [3]. However, it is still controversial whether changes in neurogenesis play a role in the pathophysiological event or act as a compensatory process in TLE. Thus, some studies indicated that newly created cells could form inappropriate neural networks increasing brain excitability which can contribute to the process of epileptogenesis [4]. On the contrary, other studies reported that the newborn cells have differentiated into inhibitory gamma-aminobutyric acid neurons, ultimately leading to reduced brain excitability [5]. Also, it has been suggested that the new cells may be involved in the regeneration of the lost neurons especially in the hippocampus, improving the memory loss following epileptic seizures. These findings suggest a compensatory role for neurogenesis in TLE [6]. Among various animal models of epilepsy, kindling is the prime choice to study the neurogenesis in TLE [7, 8]. Kindling can be induced by chemical or electrical stimulation [8]. Chemically-induced kindling is a commonly used model since it is more convenient and less expensive than electrical kindling [9]. Several reports indicated an increased proliferation during epileptogenesis in pentylenetetrazole (PTZ) fully kindled animals [10, 11]. On the other hand, only a few studies have evaluated neurogenesis during the early stages of PTZ kindling [10, 12]. This may be due to the intermingling of the seizure behaviors in the PTZ model, in contrast to electrical kindling, which makes it difficult to categorize different stages of PTZ kindling. The initial stage of seizure progression is a critical period of epileptic circuitry formation; therefore, unraveling the early behavioral stages during kindling is important for evaluating the mechanisms underlying TLE pathophysiology as well as assessing the neurogenesis in PTZ kindling [9]. Even though Racine’s scaling was commonly used in the past for classifying different stages of PTZ kindling [13]; several studies have recently suggested that it may not be sufficient for classifying PTZ kindling progress [14-16]. This is because of the observation that seizure behaviors in PTZ kindling are not identical to electrical kindling and do not occur in orders as mentioned in Racine’s scale. Besides, intermingling seizure behaviors are observed during PTZ kindling; therefore, different behavioral scoring or the numbers of PTZ injections have been proposed for assessing PTZ kindling progress [12, 14]. Although there are some improvements in new assessments: it seems that the intermingling seizure behaviors have not been completely omitted from the novel evaluations [14]. For example, when the number of PTZ injection is considered [12], various seizure behaviors may be observed with the same numbers of injections due to the various susceptibility of animals to PTZ epileptogenesis. For instance, after the fifth sub-convulsive dose of PTZ injection, some animals may show tonic-colonic behavior while others may show hind limb tonic extension or myoclonic jerk. On the other hand, cumulative scoring of different seizure behaviors [15, 17] does not seems to be a good indicator of kindling progression, since those behaviors engage different brain structures and have different electroencephalography (EEG) patterns [15]. These limitations create the demand for a valid scoring system for assessing the initial stages of PTZ kindling. Therefore, this study aimed to determine an appropriate behavioral index for categorizing stages of PTZ kindling progression and to evaluate neurogenesis during PTZ kindling. In this study, we propose using the first occurrence of seizure behaviors in mice as an index for categorizing stages of PTZ kindling progress hoping that it overcomes the current obstacles with staging PTZ kindling.

## Materials and Methods

### 1. Animals

Adult male BALB/c mice weighing 25-30g were purchased from the center of comparative and experimental medicine, Shiraz University of Medical Sciences, Shiraz, Iran. Mice were maintained under controlled temperature (22–24 °C) and lighting cycles (light on 7:00 h–19:00 h) with free access to food and water throughout the experimental period. All experiments were performed between 9:00-16:00h. Maximum effort was made to minimize the number of animals used and animals’ suffering. All procedures were performed according to the National Institutes of Health guide for the care and use of laboratory animals (NIH Publications No. 8023, revised 1978) and approved by the Ethics committee of Shiraz University of Medical Sciences (approval code: IR.Sums.REC. 1395.S890).

### 2. Stage Categorization of PTZ-Induced Kindling

Twenty-four mice were intraperitoneally injected with a sub-convulsive dose of PTZ (40mg/kg, Sigma, Germany) every other day until became full kindled [14]. Following each PTZ injection, each mouse was placed in an isolated plexiglass cage and was observed for seizure behaviors for 1 hour. Each PTZ injection elicited a relatively brief seizure behavior lasting for 20–600 seconds. In a pilot study of an ongoing project, various seizure behaviors during PTZ kindling were observed as follows: restlessness, motionless staring, hind limb tonic extension, Straub’s tail, myoclonic jerk, tonic-clonic seizures. However, these seizure behaviors did not occur in order. For instance, restlessness was usually seen after all myoclonic or tonic-clonic seizures, or it happened after all PTZ injections in all days. On the contrary, it was noticed that the first occurrence of each seizure behavior seems to occur in order during kindling progression. Therefore, we decided to examine whether the first occurrence of each seizure behavior can be used for categorizing the stages of PTZ kindling. Hence, the first occurrence of different seizure behaviors, duration of each seizure behavior when first occurred and the numbers of PTZ injections required for the first occurrence of each seizure behavior was recorded.

### 3. Evaluation of Neurogenesis During PTZ Kindling

Based on the first experiment’s results, mice (n=4-5) at each stage of PTZ kindling were selected for the evaluation of neurogenesis in SVZ. For each stage, a group of time-matched mice receiving saline was used as the control.

#### 3.1. 5-Bromo-2′-deoxyuridine (BrdU)Labeling of Proliferating Cells

Forty-eight hours after the first occurrence of the relevant seizure behavior at each stage of PTZ kindling, kindled mice and their matched controls were intraperitoneally injected with BrdU (100mg/kg, abcam, UK) four times with 2 hours’ intervals. Two hours after the last BrdU injection, animals were anesthetized via 80mg/kg ketamine and 20mg/kg xylazine (Sigma, Germany) and sacrificed by perfusing 50 mL of 4% paraformaldehyde in phosphate buffer saline (PBS, pH=7.4) via intra-cardiac puncture.

#### 3.2. Tissue Preparation

The intact brains were removed and fixed in 1% paraformaldehyde in PBS for 24 hours. Tissues were processed in an automated tissue processor, and paraffin blocks were prepared. Coronal brain sections (between bregma +0.38 mm and −0.10 mm) of 5 μm thickness were prepared along the lateral wall of the ventricle from the bottom corner to the upper corner below the corpus callosum in the lateral direction until their disappearance. Sections were then mounted on gelatin-coated glass slides. Five sections from each brain were randomly selected for immunofluorescence assay.

#### 3.3. Immunohistochemistry

The glass slides were heated and then immersed in order in xylazine, alcohol 100%, 95%, 70%, 30%, PBS, Triton X-100 at room temperature (RT), followed by immersion in HCl 2 molar at 37°C and sodium tetraborate buffer at RT. To reduce nonspecific protein bindings, the slides were incubated with blocking solution (10% normal goat serum and 0.1% bovine serum albumin in PBS) at RT for 30 min. Then, slides were incubated with mouse anti-BrdU monoclonal antibody (abcam, UK) –1:10 dilution in 10% normal goat serum, 0.1% bovine serum albumin and 1% glycine in PBS– overnight at 4 °C. After three times washing with PBS, sections were incubated with goat anti-mouse FITC-labeled secondary antibody (abcam, UK, 1:200 in dilution buffer) at RT for two hours. To stain the nuclei for calculating the total cell numbers, sections were incubated with DAPI (10µg/ml, Sigma) for 7 min at RT. Then, the cover slides were mounted with Entalan (Merck, Germany).

#### 3.4. Cell Counting

BrdU-positive cells were counted using an Olympus CX-41 microscope (Olympus Optical, Japan) equipped with a digital camera (×40 lens) and a computer-assisted image analysis system (FIJI). Two squares are selected in each section for cell counting, and the percentage of BrdU positive cells per square was calculated as follows: numbers of BrdU positive cells divided by total cell numbers and multiplied by 100. The mean percentages of BrdU positive cells per mouse sections were used for data analysis.

### 4. Statistical Analyses

Data were expressed as the mean ± standard error of the mean (SEM). Normal distribution of data was assessed by the Kolmogorov–Smirnov test. Kruskal-Wallis followed by Mann Whitney t-test was performed for comparing mean differences in the duration of seizure and neurogenesis among different stages of PTZ kindling. Mann-Whitney t-test was used for comparing mean differences in the duration of seizure and neurogenesis between each stage of PTZ kindling and its relevant time-matched control. All statistical analysis was performed using GraphPad Prism version 7.00 for Windows (GraphPad, San Diego California USA). P-value <0.05 was considered as a significant level.

## Results

### Stage Categorization of PTZ Induced Kindling

**Figure 1:**
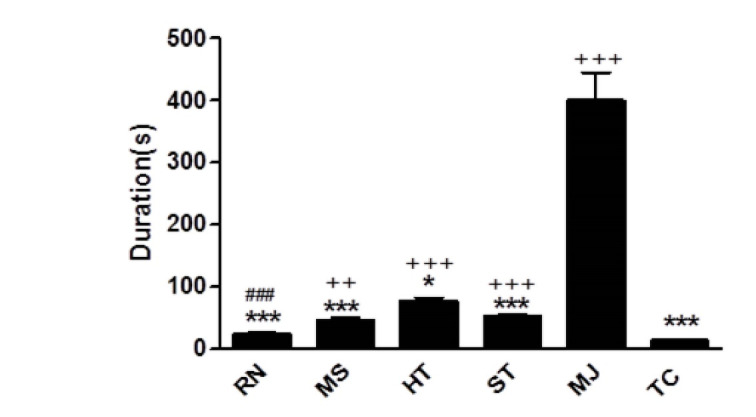
Bars represent mean + SEM of the duration of each seizure behavior in seconds (s) at their first occurrence (n=24). Restlessness (RN), motionless staring (MS), hind limb tonic extension (HT) and Straub’s tail (ST), myoclonic jerk (MJ), tonic-clonic (TC). *P<0.05 and ***P<0.001 vs. MJ, ++P<0.01 and +++P<0.001 vs. TC, ###P<0.001 vs.HT

Table-1 shows the first occurrence of each seizure behavior in reference to the highest percentage of animals showing the specific first behavior with the same number of PTZ injection. Restlessness was the first seizure behavior in 83.3% of animals with the first PTZ injection, while motionless behavior started after the second PTZ injection in 50% of mice. In many cases, restlessness and motionless behaviors simultaneously appeared after the first and second injection. The first occurrence of hind limb tonic extension and Straub’s tail were observed in 83.3% of mice after 3-4 and 3-5 PTZ injections, respectively. 83.3% of animals showed myoclonic jerk as their first seizure behavior after 6-8 PTZ injection. Tonic-clonic behavior was first noticed after 8-9 PTZ injections in 87.5% of mice. The duration of each seizure behavior when first occurred during PTZ-induced kindling was shown in Figure-1. There was a significant difference in the seizure duration among seizure behaviors (P<0.0001). Significantly, a longer duration of myoclonic jerk was observed in comparison to the duration of restlessness, motionless, Straub’s tail and tonic-clonic (P<0.001) and hind limb extension (P<0.05) behaviors. Duration of tonic-clonic behavior was significantly lower than that of motionless (P<0.01), hind limb extension and Straub’s tail behaviors (P<0.001). There was no significant difference in duration of tonic-clonic and restlessness behaviors. Duration of restlessness was significantly lower than that of hind limb extension (P<0.001). These findings show that during PTZ-induced epileptogenesis, the highest percent of animals displayed restlessness and motionless staring as their first seizure behaviors followed by hind limb tonic extension and Straub’s tail behaviors. Following higher numbers of injections, myoclonic jerk was seen just before mice became tonic-clonic with the longest duration than other seizure behaviors. Tonic-clonic, an index of full kindling, was observed with the highest numbers of injections with a lower duration than other seizure behaviors. Therefore, we classified PTZ-induced kindling into three stages including early, borderline and late phases. We defined the first occurrence of restlessness, motionless, hind limb tonic extension and Straub’s tail behaviors as an early phase, myoclonic jerk as a borderline phase and tonic-clonic as a late phase of PTZ-induced kindling.

### Neurogenesis At Different Stages of PTZ Kindling

**Figure 2:**
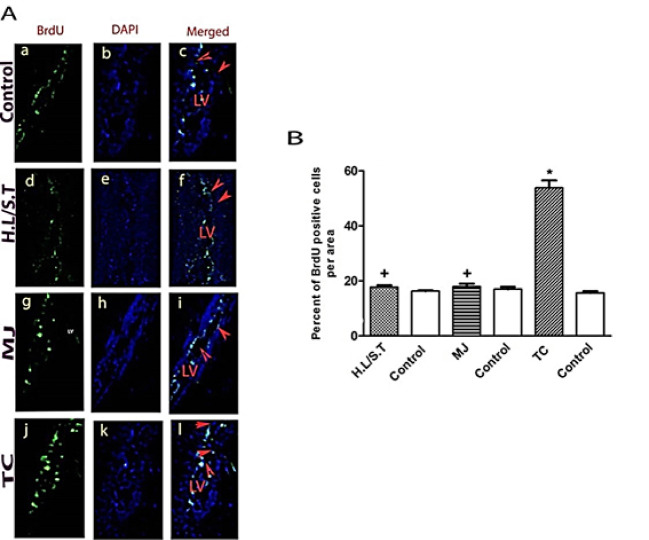
Cell proliferation during PTZ- induced kindling. A: Images of immunofluorescent double staining of sections with BrdU and DAPI were shown for control (a,b,c) H.L/S.T (d,e,f), MJ (g,h,i) and TC (j,k,l) groups. Arrows show the merged DAPI/BrdU positive cells. B: Bars represent mean + SEM of the percent of BrdU positive cells in SVZ at the different stage of PTZ kindling and their relevant time-matched groups (n=4-5 per group). Hind limb tonic extension (HT), Straub’s tail (ST), myoclonic jerk (MJ), tonic-clonic (TC). *P<0.05 vs. control, +P<0.05 vs. TC

Mann-Whitney test revealed a significantly higher percentage of BrdU-positive cells in SVZ of mice at the tonic-clonic stage in comparison to its time-matched control group (P<0.05, Figure-2). However, there were no significant differences in the percentage of BrdU-positive cells of SVZ in hind limb tonic extension/Straub’s tail and myoclonic groups as compared with their matched control groups. Also, the percentage of BrdU-positive cells in SVZ of the tonic-clonic group was significantly higher than those of hind limb tonic extension/Straub’s tail and myoclonic groups (P<0.05, Figure-2).

## Discussion

The findings of this study indicate that the first occurrence of each seizure behavior and its duration can be used to categorize different stages of PTZ-induced kindling. Based on our findings, we classified PTZ-induced kindling into three stages including early, borderline and late phases. Also, the results of this study demonstrated an enhanced SVZ neurogenesis at the late phase of PTZ-induced kindling. In the present study, a hierarchy behavior was revealed during the kindling process when considering the first occurrence of seizure behaviors. Restlessness, motionless staring, hind limb tonic extension and Straub’s tail, myoclonic jerk and tonic-clonic were observed in the order of the first occurrence. This hierarchy behavior can be attributed to more involvement of motor cortex with the progression of epileptogenesis. Hind limb tonic extension and Straub’s tail, which usually occurred simultaneously at the earliest few PTZ injections, are associated with a motor cortex region that is involved in the lower limb activity. With the higher numbers of PTZ injections and the occurrence of the myoclonic jerk, more motor cortex regions become involved including areas associated with upper limb activity and abdominal contraction. By progression of epileptogenesis and appearance of tonic-clonic, the whole motor cortex becomes involved [15, 18]. Additionally, the hierarchy behavior can be explained by different EEG patterns of seizure behaviors. Thus, increasing PTZ injections have been associated with increased EEG frequency [19]. It has been reported that motionless staring is associated with low-frequency spindle spikes; while myoclonic jerk and tonic-clonic are usually correlated with isolated spikes and multiple spikes, respectively [19]. We propose that during kindling progression, central nervous system structures become more sensitized to PTZ insult. Also, the longer neuronal activity during myoclonic jerk may stabilize brain sensitization so that when animals show myoclonic jerk, brain sensitization is at a level that further stimulation will lead to a tonic-clonic seizure. Since most of the animals, in the current study, showed myoclonic seizure with the highest duration before they became fully kindled, we recognized this stage as a borderline. This proposition should be further validated using EEG recording at different phases of PTZ kindling. Taken together, we classified PTZ-induced kindling into three phases including early, borderline and late phases. The first occurrence of restlessness, motionless, hind limb tonic extension and Straub’s tail were considered as an early, myoclonic jerk as a borderline and tonic-clonic as a late phase of PTZ kindling in mice.

Additionally, the first occurrence of each seizure behavior was scored as follows:

No seizure behavior: score 0

Restlessness: score 1

Motionless staring: score 2

Hind limb tonic extension/Straub’s tail: score 3

Myoclonic jerk: score 4

Tonic-clonic: score 5

We believe that the first occurrence of seizure behaviors can be considered as a good index for scoring and classifying PTZ-induced seizure progression. Previous studies were raising the issues regarding the use of Racin’s scoring for categorizing PTZ–induced kindling [14, 15, 20]. In agreement with our findings, Lüttjohann *et al*., study showed that the first occurrence of seizure behavior is appropriate for classifying stages of PTZ-induced epileptogenesis in rats. However, they categorized PTZ kindling into six seizure behaviors, which were different than those seen in our study [15]. This can be attributed to different seizure behaviors in rats vs. mice, as observed in our study. Therefore, for the first time, we categorized PTZ kindling progression based on the first occurrence of seizure behaviors in mice. Also, the Lüttjohann *et al*., study used rapid PTZ kindling method, while every-other-day PTZ administration schedule was used in the present study which results in slower but more consistent development of kindling [21]. In another study, PTZ kindling development was scaled based on cumulative scores of three seizure behaviors including myoclonic jerks, Straub’stail and clonus in mice [14]. Although they did not use Racine’s scoring; their scoring did not omit the issue of intermingling seizure behaviors. We have overcome this problem in the present study by considering the first occurrence of seizure behaviors and have observed a time-ordered seizure behavior during PTZ kindling. Also, more seizure behaviors such as restlessness, motionless staring and hind limb extension were assessed in our study as compared to the previous study. The results of this study indicated increased neurogenesis only at the late phase of PTZ kindling when animals showed tonic-clonic seizure. In agreement with our study, several studies are demonstrating increased neurogenesis in SVZ of full PTZ-kindled animals [10, 11]. However, there is no other report regarding neurogenesis in early or borderline stages of PTZ kindling for comparison. Nevertheless, Aniol *et al*. reported that SVZ neurogenesis decreased at the initial stage of PTZ kindling when rats did not show any seizure behaviors; and gradually increased to a normal level during kindling development following repeated PTZ injections [12]. This discrepancy could be due to using the number of PTZ injection in Aniol *et al.* study as an index of kindling progression. We believe that the number of PTZ injection is not a good index for kindling progression, because mix seizure behaviors may occur following each PTZ injection during kindling development; therefore, the resultant neurogenesis will be the outcome of various seizure behaviors with different intensities. This notion is supported by the evidence that Aniole *et al*., did not detect increased SVZ neurogenesis in PTZ full kindled animals, which is in contrast to consistent findings of other previous studies [10, 12]. In the present study, increased SVZ neurogenesis was observed at the late stage of kindling with no such alteration at early and borderline phases during kindling development. This can be explained by this concept that during kindling development with the involvement of more central nervous system structures, dramatic changes in the microenvironments of neural progenitor cells happen that can result in stimulating SVZ neurogenesis [22]. In this regard, alteration in matrix metalloproteinases-9 (MMP-9) expression may be proposed as a possible micro-environmental change that may lead to neurogenesis at late, but not the early, stage of kindling. In support of this possibility, it has been demonstrated that MMP-9 plays a vital role in the development and progression of behavioral phenotypes in PTZ kindling. Thus, in a previous study, no changes in MMP-9 were observed in rats showing facial twitching, tonic convulsion, jumping and wild running following PTZ injections. These rat behaviors are comparable to the mice behaviors that we categorized as early and borderline phases in the current study. However, repeated administration of PTZ when producing tonic-clonic or generalized seizure identical to the late phase of kindling in the present study has increased rat hippocampal MMP-9 expression. It has been shown that MMP converts pro- brain-derived neurotrophic factor (BDNF) to mature BDNF in the hippocampus [23]. BDNF has also been demonstrated to enhance proliferation of the hippocampal progenitor cells [24]. Therefore, it is likely that increased MMP-9 at the late, but not the early, phase of kindling, led to increased neurogenesis by converting pro-BDNF to mature BDNF [23].On the other hand, it is likely that changes in nitric oxide (NO) level in neural microenvironment during kindling progression, lead to increased neurogenesis at the late phase of PTZ kindling. This is supported by the evidence showing that NO plays an important role in PTZ kindling development and regulates BDNF levels in the brain [25]. Also, increased NO level and neurogenesis have been reported in fully PTZ-kindled mice, that is suggestive of NO contribution to PTZ-kindling induced neurogenesis [11]. Therefore, it may be proposed that repetitive PTZ insults during kindling may lead to a sufficient increase in NO and BDNF levels at the late, but not early, phase of kindling to enhance neurogenesis in full kindled mice. These possibilities should be further examined in future studies.

## Conclusion

In summary, the first occurrence of each seizure behavior can be used as a good index for categorizing different stages of PTZ-induced kindling. In this regard, three stages of early, borderline and late phases can be proposed. Also, our findings revealed enhanced neurogenesis only at the late phase of PTZ-induced kindling. The results of this study may help to better explain PTZ kindling progression and neurogenesis during the development of kindling. Such understandings could guide us to develop new and more effective drugs for epileptic patients.

## Acknowledgment

This study was part of a Ph.D. thesis by Marzieh Shahpari in partial fulfillment of the requirements for her Ph.D. degree in Neuroscience. The project was financially supported by a grant (#95-01-74-12015) from Shiraz University of Medical Sciences. The authors would like to thank the technical support of Ms. Fatema Pirsalami and Ms. Maryam Mojahed Taghi.

## Conflict of Interest

None.

**Table 1:** The First Occurrence of Each Seizure Behavior Was Recorded in Mice During PTZ-Induced Kindling

**First occurred Seizure Behavior**	**Percent of animal** **Showed the behavior**	**The number of PTZ injection required**
RN	83.3%	1
MS	50%	2
HT	83.3%	3-4
ST	83.3%	3-5
MJ	83.3%	6-8
TC	87.5%	8-9

**RN:** Restlessness; **MS:** Motionless staring; **HT:** Hind limb tonic extension; **ST:** Straub’s tail; **MJ:** Myoclonic jerk; **TC:** Tonic-clonic

## References

[1] Rizvi S, Ladino LD, Hernandez-Ronquillo L, Tellez-Zenteno JF (2017). Epidemiology of early stages of epilepsy: Risk of seizure recurrence after a first seizure. Seizure.

[2] Tellez-Zenteno JF, Hernandez-Ronquillo L (2012). A review of the epidemiology of temporal lobe epilepsy. Epilepsy Res Treat.

[3] Zhong Q, Ren BX, Tang FR (2016). Neurogenesis in the Hippocampus of Patients with Temporal Lobe Epilepsy. Curr Neurol Neurosci Rep.

[4] Andres-Mach M, Fike JR, Luszczki JJ (2011). Neurogenesis in the epileptic brain: a brief overview from temporal lobe epilepsy. Pharmacol Rep.

[5] Niimi Y, Levison SW (2018). Pediatric brain repair from endogenous neural stem cells of the subventricular zone. Pediatr Res.

[6] Kruger GM, Morrison SJ (2002). Brain repair by endogenous progenitors. Cell.

[7] Levesque M, Avoli M, Bernard C (2016). Animal models of temporal lobe epilepsy following systemic chemoconvulsant administration. J Neurosci Methods.

[8] Loscher W (2017). Animal Models of Seizures and Epilepsy: Past, Present, and Future Role for the Discovery of Antiseizure Drugs. Neurochem Res.

[9] Buckmaster PS (2004). Laboratory animal models of temporal lobe epilepsy. Comp Med.

[10] Park JH, Cho H, Kim H, Kim K (2006). Repeated brief epileptic seizures by pentylenetetrazole cause neurodegeneration and promote neurogenesis in discrete brain regions of freely moving adult rats. Neurosci.

[11] Zhu X, Dong J, Shen K, Bai Y, Chao J, Yao H (2016). Neuronal nitric oxide synthase contributes to pentylenetetrazole-kindling-induced hippocampal neurogenesis. Brain Res Bull.

[12] Aniol VA, Stepanichev MY, Lazareva NA, Gulyaeva NV (2011). An early decrease in cell proliferation after pentylenetetrazole-induced seizures. Epilepsy Behav.

[13] Racine RJ (1972). Modification of seizure activity by electrical stimulation II Motor seizure. Electroencephalogr Clin Neurophysiol.

[14] Dhir A. Pentylenetetrazol (PTZ) kindling model of epilepsy. Curr Protoc Neurosci 2012;Chapter 9:Unit9.37. 10.1002/0471142301.ns0937s58 23042503

[15] Luttjohann A, Fabene PF, van Luijtelaar G (2009). A revised Racine's scale for PTZ-induced seizures in rats. Epilepsy Behav.

[16] Chen Z, Li Z, Sakurai E, Izadi Mobarakeh J, Ohtsu H (2003). Chemical kindling induced by pentylenetetrazol in histamine H(1) receptor gene knockout mice (H(1)KO), histidine decarboxylase-deficient mice (HDC(-/-)) and mast cell-deficient W/W(v) mice. Brain Res.

[17] Kelly KM (2010). Aging models of acute seizures and epilepsy. Epilepsy Curr.

[18] Browning RA, Nelson DK (1986). Modification of electroshock and pentylenetetrazol seizure patterns in rats after precollicular transections. Exp Neurol.

[19] Stafstrom CE, Carmant L (2015). Seizures and epilepsy: an overview for neuroscientists. Cold Spring Harb Perspect Med.

[20] Velíšková J. Behavioral characterization of seizures in rats. In: Pitkanen A,Schwartzkroin P, Moshe S, editors. Models of seizures and epilepsy. San Diego: Elsevier Inc.; 2006.

[21] Goddard GV, McIntyre DC, Leech CK (1969). A permanent change in brain function resulting from daily electrical stimulation. Exp Neurol.

[22] Deisseroth K, Singla S, Toda H, Monje M, Palmer TD, Malenka RC (2004). Excitation-neurogenesis coupling in adult neural stem/progenitor cells. Neuron.

[23] Mizoguchi H, Yamada K (2013). Roles of matrix metalloproteinases and their targets in epileptogenesis and seizures. Clin Psychopharmacol Neurosci.

[24] Zhang Q, Liu G, Wu Y, Sha H, Zhang P, Jia J (2011). BDNF promotes EGF-induced proliferation and migration of human fetal neural stem/progenitor cells via the PI3K/Akt pathway. Molecules.

[25] Han D, Yamada K, Senzaki K, Xiong H, Nawa H, Nabeshima T (2000). Involvement of nitric oxide in pentylenetetrazole-induced kindling in rats. J Neurochem.

